# Supplementary material for: End Sequence Analysis Toolkit (ESAT) expands the extractable information from single-cell RNA-seq data
Source: Genome Res. 2016 Oct;26(10):1397–410. doi: 10.1101/gr.207902.116 (PMC5052061; doi:10.1101/gr.207902.116)
Supplement: Supplemental Material [file supp_gr.207902.116_Supp_Materials.tar.gz › ESAT-esat_v0.1/doc/html/classbroad_1_1pda_1_1seq_1_1rap_1_1_slide_and_count.html]

ESAT: broad.pda.seq.rap.SlideAndCount Class Reference


ESAT


- Main Page
- Related Pages
- Packages
- Classes
- Files

- Class List
- Class Index
- Class Hierarchy
- Class Members

All Classes Namespaces Files Functions Variables Enumerator Pages

- broad
- pda
- seq
- rap
- SlideAndCount

Static Public Member Functions |
Public Attributes |
Protected Member Functions |
List of all members

broad.pda.seq.rap.SlideAndCount Class Reference

Inheritance diagram for broad.pda.seq.rap.SlideAndCount:

[legend]

Collaboration diagram for broad.pda.seq.rap.SlideAndCount:

[legend]

|  |  |
| --- | --- |
| Static Public Member Functions | |
| static void | main (final String[] args) |
|  | |

|  |  |
| --- | --- |
| Public Attributes | |
| String | USAGE = "Slides across the genome and counts reads (or calculates ratios for two models)." |
|  | |
| Integer | WINDOW = null |
|  | |
| Integer | OVERLAP = null |
|  | |
| File | OUTPUT |
|  | |
| Public Attributes inherited from broad.pda.seq.rap.GenomeScoringProgram | |
| File | TARGET |
|  | |
| File | CONTROL = null |
|  | |
| String | SCORE = "count" |
|  | |
| boolean | PAIRED\_END =true |
|  | |
| Public Attributes inherited from broad.pda.seq.rap.GenomeCommandLineProgram | |
| String | SIZES = "/seq/lincRNA/data/mm9/sizes" |
|  | |
| String | MASK\_FILE = "/seq/mguttman/ChIPData/MaskFiles/MM9Segments/all.mask.mouse.n36.d2.bin" |
|  | |
| double | PCT\_MASKED\_ALLOWED = 50.0 |
|  | |
| String | REGION = null |
|  | |
| String | GENE = null |
|  | |
| Integer | MAX\_FRAGMENT\_LENGTH = 10000 |
|  | |
| Integer | MIN\_MAPPING\_QUALITY = 30 |
|  | |
| String | COORD\_SPACE = "genomic" |
|  | |
| String | ANNOTATION = "/seq/lincRNA/Shari/Annotations/RefSeq\_LincV3\_ChromatinWithNames\_nonrandom\_collapsed\_overlappers.bed" |
|  | |

|  |  |
| --- | --- |
| Protected Member Functions | |
| int | doWork () |
|  | |
| Protected Member Functions inherited from broad.pda.seq.rap.GenomeScoringProgram | |
| String[] | customCommandLineValidation () |
|  | |
| Protected Member Functions inherited from broad.pda.seq.rap.GenomeCommandLineProgram | |
| String[] | customCommandLineValidation () |
|  | |
| void | loadCoordinateSpace () throws IOException |
|  | |
| CoordinateSpace | getCoordinateSpace () |
|  | |

|  |  |
| --- | --- |
| Additional Inherited Members | |
| Public Member Functions inherited from broad.pda.seq.rap.GenomeScoringProgram | |
| WindowProcessor<?extends   WindowScore > | getWindowProcessor () |
|  | |
| WindowProcessor<?extends   WindowScore > | getWindowProcessor (AnnotationCollection<?extends Annotation > target, AnnotationCollection<?extends Annotation > control) |
|  | |
| Public Member Functions inherited from broad.pda.seq.rap.GenomeCommandLineProgram | |
| List< Annotation > | getRegions () |
|  | |
| Map< String, Annotation > | getRegionsMap () |
|  | |
| AnnotationList< Annotation > | getRegionSet () |
|  | |
| Annotation | getGenes () |
|  | |
| Boolean | isGenomicSpace () |
|  | |
| AlignmentModel | loadAlignmentModel (File bamFile) |
|  | |
| AlignmentModel | loadAlignmentModel (File bamFile, boolean pairedEnd) |
|  | |
| Protected Attributes inherited from broad.pda.seq.rap.GenomeCommandLineProgram | |
| CoordinateSpace | coordinateSpace |
|  | |

## Member Function Documentation

|  |  |  |  |  |
| --- | --- | --- | --- | --- |
| int broad.pda.seq.rap.SlideAndCount.doWork | ( |  | ) |  |

protected

Here is the call graph for this function:

|  |  |  |  |  |  |
| --- | --- | --- | --- | --- | --- |
| static void broad.pda.seq.rap.SlideAndCount.main | ( | final String[] | *args* | ) |  |

static

Stock main method.

Parameters
:   |  |  |
    | --- | --- |
    | args | main arguments |

## Member Data Documentation

File broad.pda.seq.rap.SlideAndCount.OUTPUT

Integer broad.pda.seq.rap.SlideAndCount.OVERLAP = null

String broad.pda.seq.rap.SlideAndCount.USAGE = "Slides across the genome and counts reads (or calculates ratios for two models)."

Integer broad.pda.seq.rap.SlideAndCount.WINDOW = null

---

The documentation for this class was generated from the following file:

- java/broad/pda/seq/rap/SlideAndCount.java


---

Generated on Fri Aug 15 2014 15:11:03 for ESAT by  

 1.8.7
